# Assessment of Water Eutrophication at Bao’an Lake in the Middle Reaches of the Yangtze River Based on Multiple Methods

**DOI:** 10.3390/ijerph20054615

**Published:** 2023-03-05

**Authors:** Mingkai Leng, Lian Feng, Xiaodong Wu, Xuguang Ge, Xiaowen Lin, Shixing Song, Rui Xu, Zhenhua Sun

**Affiliations:** 1 College of Urban and Environmental Sciences, Hubei Normal University, Huangshi 435002, China; 2 Hubei Key Laboratory of Pollutant Analysis & Reuse Technology, Hubei Normal University, Huangshi 435002, China

**Keywords:** Bao’an Lake, eutrophication assessment, comprehensive trophic level, CDOM absorption coefficient, biological method

## Abstract

Based on the monthly monitoring of Bao’an Lake in Hubei Province from 2018 to 2020, the eutrophication level of Bao’an Lake in the middle reaches of the Yangtze River is investigated using the comprehensive trophic level index (TLI), chromophoric dissolved organic matter (CDOM) absorption coefficient, and the phytoplankton water quality biological method. The influencing factors are then identified. The results demonstrate that the overall water quality of Bao’an Lake remained at levels III–V during 2018–2020. Due to different eutrophication assessment methods, the results are different, but all show that Bao’an Lake is in a eutrophication state as a whole. The eutrophication level of Bao’an Lake is observed to vary with time, exhibiting an increasing then decreasing trend between 2018–2020, while levels are high in summer and autumn, and low in winter and spring. Moreover, the eutrophication level of Bao’an Lake presents an obviously varying spatial distribution. *Potamogeton crispus* is the dominant species of the Bao’an Lake, the water quality is good in spring when *Potamogeton crispus* vigorously grows, but poor in summer and autumn. The permanganate index (COD_Mn_) and total phosphorous (TP), total nitrogen (TN), and chlorophyll a (Chl-a) contents are identified as the main influencing factors of the eutrophication level of Bao’an Lake, with a significant relationship observed between Chl-a and TP (*p* < 0.01). The above results provide a solid theoretical basis for the ecological restoration of Bao’an Lake.

## 1. Introduction

Lakes have multiple fundamental ecological functions, and the status of the lake affects regional economic and environmental security. With the growth of urban water consumption, the evolution of surface pollution, and the unreasonable discharge of sewage, the number of polluted lakes is increasing, and the water quality is deteriorating [1]. Eutrophication is the most common water environment problem in the process of water quality deterioration. Due to the large number of nutrients transported to the lake by external factors, the water begins to change from a normal nutrient state to eutrophication (a state of high productivity), which is lake eutrophication [2].

Eutrophication is often considered to be caused by the import of nitrogen and phosphorus into lakes [3,4]. Phosphorus discharged from cultivated land and concentrated in industrialization and urbanization is the key factor to promote water eutrophication [5,6]. A blue-green algae bloom is a typical case of large eutrophic lakes [7]. The massive input of nutrients accelerated the growth and reproduction of phytoplankton and promoted the outbreak of algae. The sunlight into the water gradually decreased, resulting in the death of a large number of submerged plants [8] and the intensification of lake eutrophication will affect greenhouse gas emissions [9]. In addition, the abundance of microplastics in freshwater lakes is highly correlated with the total nitrogen content, and microplastics have been proven to be one of the factors affecting the eutrophication of lakes, mainly from the tail water of urban domestic sewage treatment plants [10]. At present, scholars at home and abroad have carried out a lot of research and applied a variety of evaluation methods for eutrophication, but no standards have been established. For example, multiple methods such as the comprehensive nutritional status index method [11] and trophic state index method [12] are used to evaluate the nutritional status of water.

Although there have been a large number of studies on water quality and eutrophication assessment, there is a lack of analysis on the water quality of Bao’an Lake, a shallow lake in the southeast of Hubei Province, and few scholars have used multiple evaluation methods to comprehensively evaluate the shallow lakes in the southeast of Hubei Province. Moreover, Bao’an Lake suffers from a heavy overall pollution load, resulting in heavy pressure on its restoration and protection. Therefore, in order to achieve the effective monitoring of the Bao’an Lake water quality and restoration, the analysis and evaluation of the eutrophication level of the water body dynamics must be strengthened [13]. In the current study, the water quality status of Bao’an Lake was evaluated using TLI, the CDOM absorption coefficient, and the phytoplankton water quality biological method in order to monitor the water quality dynamics of Bao’an Lake and provide a scientific basis for the ecological restoration of the water environment.

## 2. Materials and Methods

### 2.1. Study Area

Bao’an Lake is located in southeastern Hubei Province (30°12′ N–30°20′ N, 114°40′–114°48′ E), in the northwestern part of Daye City, at the east of Sanshan Lake, west of Liangzi Lake, south of Bao’an Town, and north of Dongfeng Farm [14]. It is a typical shallow lake of the Liangzi Lake Group on the south bank of the middle reaches of the Yangtze River [15]. The watershed area is 285 km^2^, the lake area is 39.3 km^2^, the maximum water depth is 3.7 m, and the average water depth is 1.5–2.5 m [16]. Bao’an Lake was previously connected to Liangzi Lake, but now the two lakes are separated, and the water level is regulated through Donggou Gate. The flow direction of the Bao’an Lake system is from south to north, and the runoff mainly originates from the Huandiqiaogang River, the Bao’andong River, and the Bao’anxi River, while the outflow mainly flows from the Donggou Port in the northwest into Changgang, and subsequently discharges into the Yangtze River through the Fankou Dam [17].

### 2.2. Sampling Site Arrangement and Index Measurements

**Figure 1:**
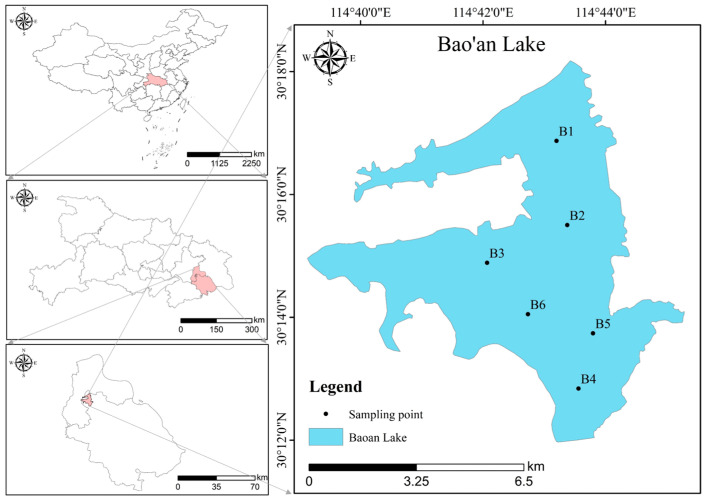
Distribution of sampling sites in Bao’an Lake.

Six sampling sites were arranged according to the area and water surface shape of Bao’an Lake (Figure 1). In order to understand the trend of change of the water body of Bao’an Lake within and between years, samples were collected from January 2018 to December 2020 from a monthly survey. Water samples were collected at 0.5 m from the water surface using a plexiglass water collector. The water samples were filled in 2.5 L acid-washed polyethylene bottles and quickly brought back to the laboratory for the analysis of the water quality indicators.

Depth and SD were measured in situ during the water sample collection using a bathymetry instrument (SM-5A, Speedtech) and a 30 cm Secchi disk, respectively [18]. TN and TP were measured using potassium persulfate oxidation—ultraviolet spectrophotometry and molybdenum-antimony anti-spectrophotometry, respectively. NH_3_-N was measured using Nessler’s reagent spectrophotometry, COD_Mn_ was measured using the acid method, and Chl-a was measured using the 90% acetone extraction method. Water temperature, dissolved oxygen (DO), and pH were measured by using the YSI EXO2 multi-parameter water quality monitor [19,20].

The absorption coefficient of CDOM was determined by a GF/C filter membrane with 0.45 μm and 0.22 μm pore diameter. Phytoplankton samples were collected with reference to the standard method [21].

### 2.3. Data Processing and Evaluation Methods

ArcGIS 10.2 (Esri, Redlands, California, USA) was used to map the distribution of the sampling sites and perform spatial interpolation analysis. Excel 2019 (Microsoft Corp, Redmond, WA, USA) and SPSS 24.0 (IBM, Armonk, NY, USA) were employed to analyze and process the data, and Origin 2019 (OriginLab) was used to complete the mapping.

#### 2.3.1. TLI

According to the characteristics of Bao’an Lake, the TLI recommended by the China National Environmental Monitoring Station was adopted. TLI is an indicator of the comprehensive eutrophication level and is used to evaluate water body quality by integrating representative water quality parameters, including Chl-a, SD, TP, TN, and COD_Mn_ [22]. The TLI final value is calculated by multiplying the trophic level indices with their corresponding weights. The comprehensive trophic level index (TLI (Σ)) can be calculated as:

(1) $$\mathrm{TLI}(\sum )=\sum_{j=1}^{m}W_{j}\cdot \mathrm{TLI}(j),\;$$

where TLI (Σ) is the comprehensive trophic level index; W_j_ is the jth weight value of the trophic level index; and TLI (j) is the jth trophic level index [23].

With Chl-a as the base parameter, the normalized correlation weight of the jth parameter can be calculated as follows:

(2) $$W_{j}=\frac{r_{\mathrm{ij}}^{2}}{\sum_{j=1}^{m}r_{\mathrm{ij}}^{2}}$$

where r_ij_ is the correlation coefficient between the jth parameter and the benchmark parameter Chl-a; and m is the number of evaluation parameters. The trophic level index of all indicators can be calculated via Equations (3)–(7) [20] and used to grade the lake trophic level using Table S1.

(3) $$\mathrm{TLI}\left(\mathrm{Chl}\text{-}a\right)=10(2.5+1.086\mathrm{lnChl}\text{-}a),\;$$

(4) $$\mathrm{TLI}\left(\mathrm{TP}\right)=10(9.436+1.624\mathrm{lnTP}),\;$$

(5) $$\mathrm{TLI}\left(\mathrm{TN}\right)=10(5.453+1.694\mathrm{lnTN}),\;$$

(6) $$\mathrm{TLI}\left(\mathrm{SD}\right)=10(5.118-1.940\mathrm{lnSD}),\;$$

(7) $$\mathrm{TLI}\left({\mathrm{COD}}_{\mathrm{Mn}}\right)=10(0.109+2{.661\mathrm{lnCOD}}_{\mathrm{Mn}}).\;$$

#### 2.3.2. Chromophoric Dissolved Organic Matter (CDOM) Absorption Coefficient Method

The eutrophication status of Bao’an Lake is evaluated according to the “Assessment Method of Lake Eutrophication based on the Absorption Coefficient of Colored Soluble Organic Matter” proposed by Zhang Yunlin and others.

Water samples were collected from six sites of Bao’an Lake on 17 January, 25 April, 18 July, and 10 October 2019. The absorption spectrum of CDOM was measured by ultraviolet-visible spectrophotometry (UV2700) with a cuvette range of 0.01 m. The absorbance of the sample was scanned at 1 nm intervals from 200 nm to 800 nm using ultrapure water as a reference. The absorption coefficient can be calculated as follows [24]:

*A* (λ′) = 2.303 *A* (λ)/*r*, (8)

where *A* (λ′) is the uncorrected absorption coefficient at λ, m^−1^; *A* (λ) is the absorption coefficient at λ, abs; and *r* is the optical range of the cuvette, m. Because some fine particles remain in the filtrate and affect the absorption measurement, the absorption coefficient is corrected for scattering, usually at 700 nm as follows [25]:

*a* (λ) = *a* (λ′) − *a* (700) λ/700, (9)

where *a* (λ) is the corrected absorption coefficient at λ, m^−1^ and and λ is the wavelength, nm [26].

The absorption coefficient at 254 nm was determined using Equation (8) and the scattering correction was performed following Equation (9) to obtain the corrected absorption coefficient at 254 nm. The trophic level of the lake was classified according to the measured absorption coefficient a(254) of CDOM at 254 nm: a(254) < 4 for oligotrophication; 4 ≤ a(254) ≤ 9 for medium; and a(254) > 9 for eutrophication, where 9 < a(254) ≤ 15 is mild eutrophication, 15 < a(254) ≤ 22 is moderate eutrophication, and a(254) > 22 is severe eutrophication [27].

#### 2.3.3. Phytoplankton Water Quality Biological Method

Current biological evaluations of water quality are generally based on phytoplankton density, diversity index, and other indicators. The biological species of water bodies with different water quality and nutrient levels are different. The phytoplankton cell density, Shannon–Wiener diversity index (H′) [28], Margalef abundance index (D′) [29], and Pielou evenness index (J′) [30] are linked to water quality [31]. Hence, cell density, H′, J′, and D′ were used to evaluate the nutritional status of Bao’an Lake [32]. Table S2 reports the trophic level diversity index criteria.

## 3. Results and Analysis

### 3.1. Current Status of Physical and Chemical Indicators of Lake Water Bodies

The water quality monitoring results of Bao’an Lake reveal that the water quality indicators greatly fluctuate from season to season. In particular, DO concentrations were minimized in autumn, while the three-year average value of SD was the highest in spring and the lowest in autumn. The maximum mean COD_Mn_ concentration was observed in autumn. TP and TN followed the same seasonal trends, with slightly higher concentrations in spring and autumn. Chl-a concentrations were highest in summer and lowest in spring, with the former about 2.39 times higher.

**Figure 2:**
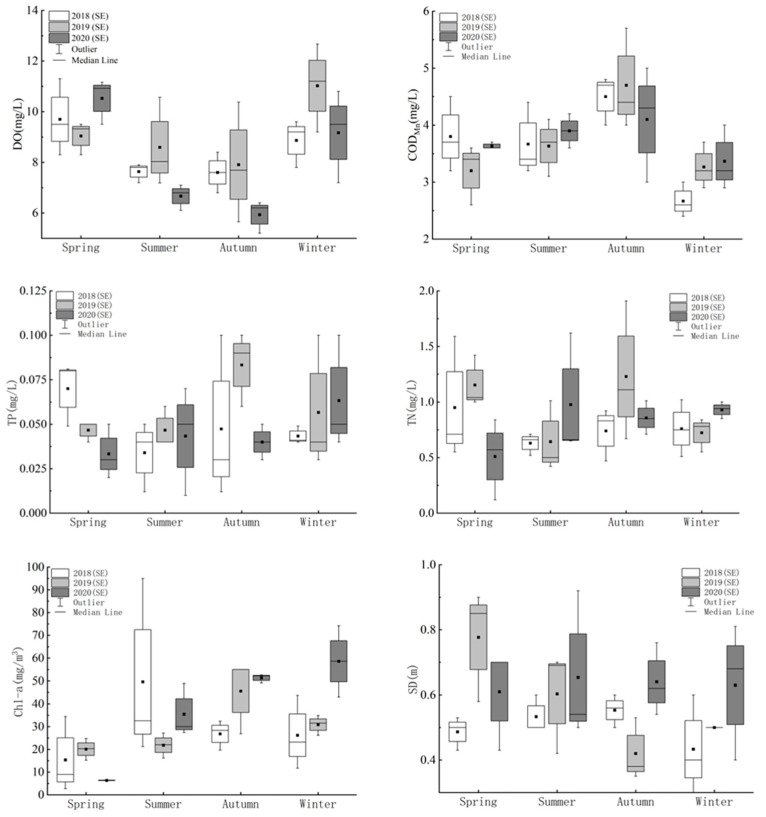
Seasonal variation in water quality indicators.

The average concentration of DO was 8.55 ± 1.46 mg/L and pH was 7.9 ± 0.22. The SD fluctuated between 0.42 and 0.92 m with an average of 0.58 m (Figure 2). The mean COD_Mn_ and TP concentrations were 3.70 ± 0.70 mg/L and 0.049 ± 0.02 mg/L, respectively, with the latter indicating class V water. In addition, the TN content was 0.84 ± 0.36 mg/L, indicating class III water, and the mean NH_3_-N was 0.283 ± 0.17 mg/L, indicating class II water. The Chl-a concentration was determined as 29.48 ± 22.70 mg/m^3^, with a maximum value of 94.94 mg/m^3^.

### 3.2. Comprehensive Trophic Level Index Assessment

The assessment of the comprehensive trophic level index reveals that the eutrophication level of Bao’an Lake remained at medium to low in 2018–2020 (Table 1), with a small interannual variation (51.68–54.79), and an overall increasing and subsequent decreasing trend. The comprehensive trophic level index reached its lowest value in 2018–2020 in winter (50.12), summer (49.76), and spring (41.28), respectively.

### 3.3. CDOM Absorption Coefficient Assessment

The results of the CDOM absorption coefficient indicate the eutrophication level of Bao’an Lake to remain low during the entire year. The water quality was poor in summer, with a consistently moderate eutrophication level (Table 2). The range of the absorption coefficient variation at 254 nm was small at each site, from 7.176 m^−1^ to 17.189 m^−1^ throughout 2019. In terms of seasonal variation, the absorption coefficient peaked in summer, with all sites except site 1 exceeding 15 m^−1^ and an average of 17.014 m^−1^. The absorption coefficient ranged from 11–12 m^−1^ in spring, autumn, and winter.

**Figure 3:**
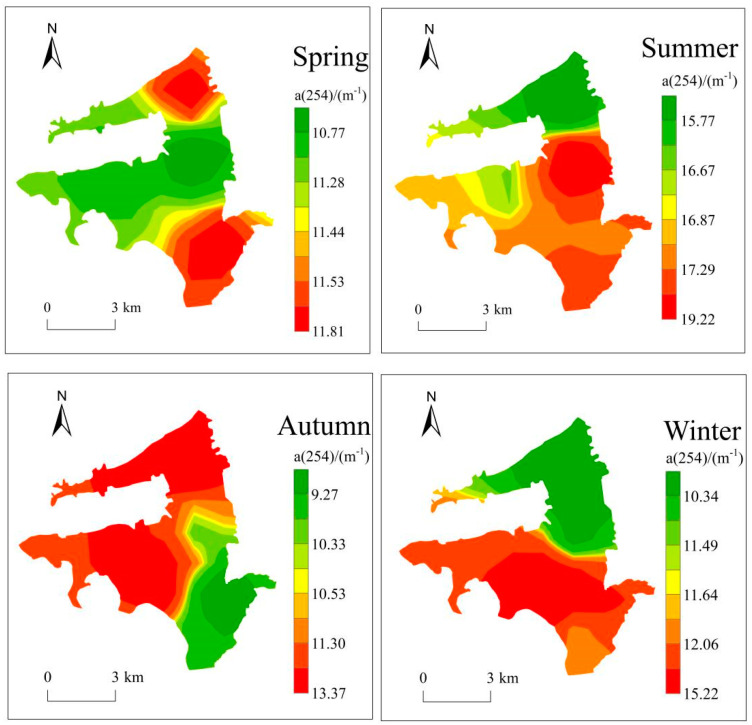
Spatial–temporal trends of a(254) in Bao’an Lake by season.

Inverse distance weight (IDW) interpolation was conducted using ArcGIS based on the 254 nm CDOM absorption coefficient from all sites in Bao’an Lake in order to retrieve the seasonal spatial distribution of a(254) in the lake. This represents the spatial distribution of the lake eutrophication level (Figure 3). The spatial distribution of a(254) was observed to vary among seasons, with high and low values interspersed in spring and summer, and the lowest value in the central-northern part of the lake in spring. Furthermore, a(254) values in spring were generally high in the northern and southern regions and low in the center. In summer, values peaked in the central-northern part of the study area, and the overall pattern was high in the south and low in the north. There is a clear distribution of high and low values in autumn and winter. In particular, the lowest values in autumn were observed in the central-southern part of Bao’an Lake, while values were generally high in the northwest and low in the southeast. In winter, a(254) values did not exceed 10 m^−1^ in B1 and B2 within the northern part of the lake, while in the southern part of the lake, a(254) was consistently higher than 11 m^−1^ in all four sampling sites.

### 3.4. Biological Assessment Based on Phytoplankton Water Quality

#### 3.4.1. Assessment Based on Algae Cell Density

The evaluation standards for algae cell density revealed Bao’an Lake to have a moderate eutrophication level throughout the year, with algae cell density fluctuating within the range of 270.67 × 10^4^ to 6138.13 × 10^4^ cells·L^−1^. In 2019 and 2020, the algae cell density was maximum in spring and autumn, with lower eutrophication levels in spring (low-medium) and an algae cell density range of 633.97 × 10^4^ and 270.67 × 10^4^ cells·L^−1^, respectively. The eutrophication level was slightly higher in autumn, with an algae cell density of 6138.13 × 10^4^ cells·L^−1^ (medium eutrophication level) in the autumn of 2019 (Table 3).

#### 3.4.2. Phytoplankton Diversity Index Assessment

The evaluation results of the phytoplankton diversity index reveal the eutrophication of Bao’an Lake as medium, with a moderate water pollution level (Table 4). The mean value of the Shannon–Wiener index was 2.12, with the highest value (2.64) at site B2 and the lowest (0.99, approximately 0.375 times that of the highest value) at B1. In addition, the mean value of the Pielou index was 0.45, with the highest value of 0.53 (B6), equal to approximately 2.30 times that of the lowest value (B1). The mean Margalef index value was determined as 1.35, and all sites exhibited a eutrophication level.

## 4. Discussion

### 4.1. Identification of Factors Influencing Water Eutrophication

Principal component analysis (PCA) was conducted on nine water quality indexes (WT, pH, DO, COD_Mn_, NH_3_-N, TP, TN, Chl-a, and SD) [33]. Following KMO (Kaiser–Meyer–Olkin) and Bartlett’s Sphericity tests and stepwise screening at KMO > 0.5, the final KMO value was determined as 0.657 and *p* = 0.045 (*p* < 0.05) when only five indicators of Chl-a, COD_Mn_, TP, TN, and SD were used. The results indicate that the parametric data could be used for PCA. Based on the principle that the eigenvalues should be greater than 1, two principal components were extracted, with a variance contribution of 54.189% and 25.198%, respectively, and a cumulative contribution of 79.387% (Table S3).

The first principal component had a large factor loading with COD_Mn_, TP, TN, and Chl-a (R_Chl-a_ = 0.884; R_TP_ = 0.866; R_TN_ = 0.786; R_CODMn_ = 0.741) (Table S4). This suggests that the first principal component mainly reflects the chemical indicators affecting the water quality factors, while N, P [34], and COD_Mn_ [35] can promote the growth of phytoplankton in the water body, and Chl-a indirectly reflects the growth of phytoplankton in the water body [36]. The second principal component had a large factor loading (R_SD_ = 0.957) with the physical index SD, which can visually reflect the pollution status of the water body [11].

The analysis results imply that COD_Mn_, Chl-a, TN, TP, and SD are the dominant influencing factors of the Bao’an Lake eutrophication, This agrees with the work of Zhang Yanyan’s research on the leading factors of eutrophication in Dishui Lake, Beili Lake, and Yinhoe Lake in Zhejiang Province and Shanghai, China [37].

Chl-a can reflect the growth of planktonic algae in the lake body [38], and nitrogen and phosphorus are closely related to the trophic level of water bodies [39]. Therefore, the correlation of Chl-a with TN and TP was selected to reflect the influence of planktonic algal biomass on the nutrient status of the lake water body. The level of Chl-a, TN, TP, and other indicators from Bao’an Lake were standardized and Pearson’s correlation analysis was performed (Table S5). The results showed that COD_Mn_ and Chl-a were significantly correlated (*p* = 0.043, *p* < 0.05), and TP and Chl-a were highly significantly correlated (*p* = 0.008, *p* < 0.01), while SD and NH3-N were less correlated with the rest of the indicators. Moreover, TP and TN were significantly correlated (*p* = 0.037, *p* < 0.05). The final main influencing factors of the Bao’an Lake eutrophication were determined to be COD_Mn_, TP, TN, and Chl-a. This is consistent with previous work on the main driving factors of the eutrophication of Gao Yang Ping Lake in the Three Gorges reservoir area in China [40]. Phosphorus control is crucial to alleviate eutrophication. In particular, phosphorus carried by farmland runoff is an important component of non-point source pollution. At the same time, the synergism of nitrogen and phosphorus will lead to the evolution of the water nutrient state to eutrophication [41,42]. Therefore, the reduction of TP and TN input is the most important means to restore water eutrophication [43].

### 4.2. Effects of Basin Land Utilization on Bao’an Lake Water Quality

The main land types within the Bao’an Lake basin are agricultural land and construction land, of which agricultural land typically includes intensive fishponds and arable land. Potential associations have been reported between the land use type and the water quality of rivers and lakes [44]. Farmland, fish ponds, and construction sites generally lead to the deterioration of water quality in rivers and lakes [45], while woodlands and grasslands in agricultural land play an active role in maintaining water quality [46].

The area of farmland and fishponds in the Bao’an Lake watershed is relatively large, reaching 71.18% of the total basin. Forest land, grassland, water bodies, and construction land account for 6.19%, 0.013%, 14.11%, and 8.48% of the basin area of Bao’an Lake, respectively [47]. The results of the field survey showed that the fishponds on the northwest side of the Bao’an Lake covered an area of about 400 Mu, most of which were intensive fishponds in fertilization mode (Figure S1). Regular water exchange in the fishponds will result in the discharge of farming wastewater into Bao’an Lake. Furthermore, there is a large fishpond around the northern part of Bao’an Lake, which is also regularly exchanged with the Bao’an Lake water body. The fertilized fishponds on the eastern bank of the Bao’an Lake have a size of 340 Mu, with a greenish color and serious eutrophication. The wastewater can be directly channeled into the Bao’an Lake through western culvert pipes, severely threatening the water quality of the lake. In addition to being a source of water for agricultural irrigation, the river and its tributaries are also used for the drainage of agricultural fields, and it is still common for agricultural ditches to directly discharge into the river. Moreover, the sewage network around Bao’an Lake is not yet complete, resulting in a small amount of sewage still being discharged into the lake.

The accumulation of nutrient salts such as nitrogen and phosphorus caused by irrigation receding water from agricultural fields and fish pond tailwater [13], as well as the accumulation of pollutants on impervious surfaces in towns [48], will increase the pollution level of the water body when it flows into Bao’an Lake with the incoming rivers.

The assessment of the CDOM absorption coefficient reveals the high comprehensive eutrophication index values to be concentrated in the eastern and southern areas, where there is a large amount of construction land.

### 4.3. Effects of Submerged Plant Distribution on the Water Quality of Bao’an Lake

Submerged plants can effectively reduce water turbidity [49] and significantly improve water quality indicators such as Chl-a, TN, and TP [50]. Furthermore, the overgrowth or disappearance of submerged plants can also adversely affect lake ecosystems [51]. In the mid-20th century, Bao’an Lake was rich in water and grasses and had more than 50 species of aquatic vegetation. By 2003, submerged plants covered 92.9% of the lake area, with *Ceratophyllum demersum*, *Myriophyllum spicatum*, *Potamogeton maackianus,* and *Vallisneria natans* as the dominant species, and the emergent vegetation and leave-floating plants zones were essentially extinct [52]. In recent years, with the increase of the eutrophication level and artificial ponds around the lake, the aquatic vegetation has been reduced, while *Potamogeton crispus* gradually adapted to the eutrophicated water environment to become the dominant species of aquatic vegetation in Bao’an Lake [53]. In 2019, *Potamogeton crispus* was the most distributed and abundant species in the central part of Bao’an Lake, with a maximum biomass of 3916 g/m^2^ at a single sampling site. The water quality in areas with high coverage of *Potamogeton crispus* was obviously better than that in open water areas [16].

*Potamogeton crispus* has a purifying effect on phosphorus in water bodies [54], and during its growth, it absorbs nutrients from the water body and stores them in the plant or transports them to the sediment. Moreover, photosynthesis of *Potamogeton crispus* was also reported to significantly increase the DO and pH of the water [55]. This species typically dies in late spring, and the nitrogen and phosphorus elements in the decaying *Potamogeton crispus* re-enter the water body causing further deterioration. Following this, the corrupted plants under the water’s surface will release organic phosphorus, providing favorable environmental conditions for the growth of algae and further causing its bloom [56]. Previous research on phosphorus release during the decomposition of the *Potamogeton crispus* reported TP, TN, and COD_Mn_ in the water body to be maximized on day 16 after the death of *Potamogeton crispus*, followed by a rapid decline until day 45. This demonstrates the short-duration effect of *Potamogeton crispus* on the water quality of Bao’an Lake [57].

Due to the low variety of aquatic vegetation in Bao’an Lake and its fragile ecosystem, the water quality is better in spring when *Potamogeton crispus* is actively growing, and is relatively poor during the summer and autumn when *Potamogeton crispus* is declining and dormant. Thus, the eutrophication level is higher in summer and autumn compared to spring.

### 4.4. Assessment of Phytoplankton Diversity and Water Quality

Phytoplankton occupies an important niche in aquatic ecosystems and is a major food source for aquatic animals [58]. It is also highly sensitive to environmental changes [59], which can serve as an indicator of water quality [60]. Therefore, changes in the structure of phytoplankton communities can cause significant changes in the trophic level of water bodies [61]. In addition, the water quality of a lake is affected by various external factors, including soil characteristics, vegetation, runoff, and surrounding land use practices. Protecting the ecological functions of lakes is essential to maintain water quality, and changes in phytoplankton community structure should be considered while controlling nutrient and other pollutant inputs.

Biodiversity is an important indicator of the ecological characteristics of phytoplankton communities. It can not only be used to evaluate the degree of water pollution, but also reflect the stability of water from the ecosystem level [62]. In the current study, the assessment based on the phytoplankton diversity index indicates a medium eutrophication level for Bao’an Lake, while the algae cell density method and diversity index suggest contrasting results. This may be because Bao’an Lake is a grass-algae type lake, where *Potamogeton crispus* grows and reproduces from autumn to spring and declines in late spring and early summer. Thus, Bao’an Lake is a grass-type lake in winter and spring, with relatively low phytoplankton biomass and medium or even low-medium eutrophication. However, in summer and autumn, it is an algae-type lake with a high water eutrophication level. The distribution of Bao’an Lake phytoplankton was observed to be uneven throughout the lake area, with a clear downward trend from north to south. The minimum Shannon–Wiener, Pielou, and Margalef index values were determined for the northern part of Bao’an Lake, at 0.99, 0.23, and 0.96, respectively, all of which corresponded to high eutrophication levels. Moreover, the diversity index of the sampling sites significantly varied, also influencing the evaluation results.

## 5. Conclusions

Based on the application of different methods, this study revealed that the eutrophication level of Bao’an Lake is essentially low (TLI: 41.28~57.98) and obviously varies with time and space. From 2018 to 2020, the eutrophication level increased first and then decreased with the seasons, and the TLI reached the highest value in autumn (51.18, 57.98, 53.13). According to the evaluation of the CDOM absorption coefficient, the eutrophication level of Bao’an Lake was higher in the south (mean: 13.14) and lower (mean: 12.39) in the north. *Potamogeton crispus* is the dominant species of Bao’an Lake. The water quality is good in spring when *Potamogeton crispus* vigorously grows (TLI: 41.28~49.97) and is poor in summer and autumn due to the decay period of *Potamogeton crispus* (TLI: 49.76~57.98). Bao’an Lake also exhibits low phytoplankton biomass and large differences in the diversity index (H′: 2.12; J′: 0.45; D′: 1.35). In particular, the assessment results of algae cell density and diversity index are different. Furthermore, intensive fishponds, agriculture, and construction are the major causes of the water eutrophication of Bao’an Lake. Follow-up treatments should focus on the improvement of intensive fishponds and cultivated land patterns, as well as the restoration of aquatic vegetation, in order to inhibit the growth and reproduction of algae and comprehensively improve the quality of the water environment.

## Figures and Tables

**Table 1:** Assessment of waterbodies by the trophic level comprehensive index.

Year	Season	TLI(Chl-a)	TLI(TP)	TLI(TN)	TLI(SD)	TLI(COD_Mn_)	TLI(∑)	Eutrophication Level
2018	Spring	49.34	50.76	51.84	65.23	36.35	50.58	Light eutrophication
	Summer	65.15	36.78	46.56	63.45	35.40	50.72	Light eutrophication
	Autumn	60.48	38.97	48.74	62.72	41.03	51.18	Light eutrophication
	Winter	58.97	43.32	49.22	68.19	27.08	50.12	Light eutrophication
2019	Spring	57.37	44.50	56.73	56.43	31.78	49.97	Mesotrophication
	Summer	58.23	44.28	45.77	61.50	35.25	49.76	Mesotrophication
	Autumn	65.92	53.63	56.51	68.33	41.97	57.98	Light eutrophication
	Winter	62.17	45.49	48.77	64.63	32.46	51.64	Light eutrophication
2020	Spring	45.07	34.12	31.81	58.10	35.54	41.28	Mesotrophication
	Summer	63.38	38.82	52.48	60.19	37.25	51.43	Light eutrophication
	Autumn	67.76	41.74	51.73	60.03	38.05	53.13	Light eutrophication
	Winter	68.81	43.90	52.63	56.96	33.70	52.62	Light eutrophication

**Table 2:** Absorption coefficient of CDOM at 254 nm in different seasons of Bao’an Lake and corresponding evaluation results.

Site	Spring a(254) (m^−1^)	Summer a(254) (m^−1^)	Autumn a(254) (m^−1^)	Winter a(254) (m^−1^)
B1	11.892	14.195	13.561	7.830
B2	10.050	19.639	9.897	9.275
B3	10.741	16.435	12.180	12.960
B4	11.662	17.586	9.436	11.264
B5	11.892	17.042	7.176	13.588
B6	11.431	17.189	13.665	15.807
Total lake Eutrophication level	Light eutrophication	Moderate eutrophication	Light eutrophication	Light eutrophication

**Table 3:** Algae cell density and eutrophication status of Bao’an Lake.

Year	Season	Algae Cell Density (10^4^ Cells·L^−1^)	Eutrophication Level
2019	Spring	633.97	Oligotrophication-Mesotrophication
	Summer	1279.63	Mesotrophication
	Autumn	6138.13	Mesotrophication-Eutrophication
	Winter	1609.20	Mesotrophication
2020	Spring	270.67	Oligotrophication-Mesotrophication
	Summer	2141.43	Mesotrophication
	Autumn	3216.17	Mesotrophication
	Winter	2860.50	Mesotrophication

**Table 4:** Phytoplankton diversity index and result.

Assessment Index	B1	B2	B3	B4	B5	B6	Mean Value	Status	Qualitative Evaluation
Shannon–Wiener (H′)	0.99	2.64	2.29	2.13	2.27	2.38	2.12	Mesotrophication	Slight pollution
Pielou (J′)	0.23	0.48	0.50	0.43	0.51	0.53	0.45	Mesotrophication-Eutrophication	Moderate pollution
Margalef (D′)	0.96	2.32	1.15	1.46	1.10	1.11	1.35	Eutrophication	Heavy pollution

## Data Availability

The datasets generated and/or analyzed during the current study are available from the corresponding author on request.

## Supplementary Materials

The following supporting information can be downloaded at: https://www.mdpi.com/article/10.3390/ijerph20054615/s1, Figure S1: Distribution of intensive fishponds surrounding Bao’an Lake; Table S1: Lake trophic level grading table; Table S2: Diversity index and criteria for the evaluation of the trophic level of water bodies; Table S3: PCA Variance analysis table of eutrophication influencing factors; Table S4: PCA rotation matrix of eutrophication influencing factors; Table S5: Correlation analysis of water quality factors of Bao’an Lake.
