# Supplementary material for: Assessment of Water Eutrophication at Bao’an Lake in the Middle Reaches of the Yangtze River Based on Multiple Methods
Source: Int J Environ Res Public Health. 2023 Mar 5;20(5):4615. doi: 10.3390/ijerph20054615 (PMC10002378; doi:10.3390/ijerph20054615)
Supplement: Supplementary file 1 [file ijerph-20-04615-s001.zip › ijerph-2246939-supplementary.pdf]

Supplementary Materials for

Assessment of water eutrophication at Bao'An Lake in the middle reaches of the Yangtze River based on multiple methods

Figs. S1

Tables S1 to S5

Table S1. Lake trophic level grading table

Trophic level	Evaluation criteria: TLI(∑)	Qualitative evaluation
Oligotrophication	TLI(∑)<30	Great
Mesotrophication	30≤TLI(∑)≤50	Good
Eutrophication	TLI(∑)>50	Polluted
Light eutrophication	50<TLI(∑)≤60	Lightly polluted
Moderate eutrophication	60<TLI(∑)≤70	Moderately polluted
Heavy eutrophication	TLI(∑)>70	Heavily polluted

Table S2. Diversity index and criteria for the evaluation of the trophic level of water bodies

Trophic level	Cell density (10^4 cells·L^{-1})	Diversity index (H′)	Evenness index (J′)	Abundance index (D′)	Qualitative evaluation
Extreme oligotrophication	50<	>3	>0.8	>5	Great
Oligotrophication	50-100				
Oligotrophication-Mesotrophication	100-900				
Mesotrophication	900-4,000	2~3	0.5~0.8	4~5	Lightly polluted
Mesotrophication-Eutrophication	4,000-8,000	1~2	0.3~0.5	3~4	Moderately polluted
Eutrophication	8,000-10,000	0~1	0~0.3	0~3	Heavily polluted

Extreme Eutrophication	>10,000

Table S3. PCA Variance analysis table of eutrophication influencing factors

Factor	Initial eigenvalues			Sum of squared loading		
	Sum	Percentage of variation	Accumulation %	Sum	Percentage of variation	Accumulation %
1	2.709	54.189	54.189	2.709	54.189	54.189
2	1.260	25.198	79.387	1.260	25.198	79.387
3	0.516	10.327	89.714			
4	0.308	6.165	95.880			
5	0.206	4.120	100.000			

Table S4. PCA rotation matrix of eutrophication influencing factors

Monitored factors	Component 1	Component 2
COD_{Mn}	0.741	0.429
TP	0.866	-0.225
TN	0.786	0.274
Chl-a	0.884	-0.206
SD	-0.046	0.957

Table S5. Correlation analysis of water quality factors of Bao'an Lake

Index	WT	pH	DO	COD_{Mn}	NH_3-N	TP	TN	Chl-a	SD
WT	1								
pH	-0.493	1							
DO	-0.267	0.289	1						
COD_{Mn}	.630*	-.587*	-0.520	1					
NH_3-N	0.489	-0.575	-0.242	0.323	1				
TP	0.290	-0.525	-0.080	0.466	0.021	1			
TN	0.293	-0.573	-0.127	0.500	.661*	.604*	1		
Chl-a	0.411	-0.256	-0.281	.628*	0.084	.725**	0.517	1	
SD	0.476	-0.488	0.363	0.184	0.533	-0.152	0.215	-0.200	1

*** denotes significance at the 0.05 level (two-tailed); ** denotes high significance at the 0.01 level (two-tailed).**

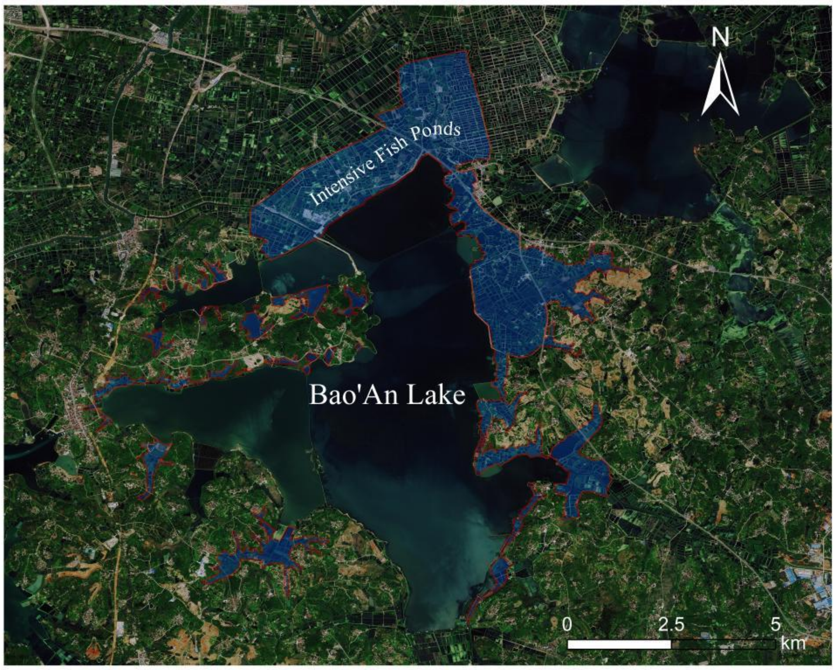

Figure S1. Distribution of intensive fishponds surrounding Bao'An Lake
